# Leveraging Human Perception in Robot Grasping and Manipulation Through Crowdsourcing and Gamification

**DOI:** 10.3389/frobt.2021.652760

**Published:** 2021-04-29

**Authors:** Gal Gorjup, Lucas Gerez, Minas Liarokapis

**Affiliations:** New Dexterity Research Group, Department of Mechanical Engineering, The University of Auckland, Auckland, New Zealand

**Keywords:** crowdsourcing, gamification, grasping, robot perception, image classification

## Abstract

Robot grasping in unstructured and dynamic environments is heavily dependent on the object attributes. Although Deep Learning approaches have delivered exceptional performance in robot perception, human perception and reasoning are still superior in processing novel object classes. Furthermore, training such models requires large, difficult to obtain datasets. This work combines crowdsourcing and gamification to leverage human intelligence, enhancing the object recognition and attribute estimation processes of robot grasping. The framework employs an attribute matching system that encodes visual information into an online puzzle game, utilizing the collective intelligence of players to expand the attribute database and react to real-time perception conflicts. The framework is deployed and evaluated in two proof-of-concept applications: enhancing the control of a robotic exoskeleton glove and improving object identification for autonomous robot grasping. In addition, a model for estimating the framework response time is proposed. The obtained results demonstrate that the framework is capable of rapid adaptation to novel object classes, based purely on visual information and human experience.

## 1. Introduction

Over the last decades, autonomous intelligent robotic systems have achieved notable levels of speed, precision, and repeatability, surpassing human ability to execute a wide range of tasks that involve some form of interaction with the environment. Despite the significant progress in the robot grasping and manipulation fields (Mahler et al., 2017), humans still excel in activities related to perception and reasoning due to the complexity, subjectivity, and uncertainty involved in these processes (Torresen, 2018). In order for robots to better understand and react to changes in their surroundings, they need environmental awareness and intelligent reasoning that will lead to sophisticated problem-solving. This is not yet feasible with traditional artificial intelligence methods, but can be achieved by involving humans in the decision-making process.

Recently, robotics researchers started utilizing human intelligence through crowdsourcing to solve complex tasks, improving the capabilities of existing autonomous intelligent systems (Chernova et al., 2010; Breazeal et al., 2013; Kehoe et al., 2015; Zhao and Han, 2016; Ibáñez et al., 2020). For instance, in Gouravajhala et al. (2018), the authors propose a system that employs online non-expert human crowds to cooperate with robots to segment and label objects in 3D point clouds. Through the system, a single worker was able to segment a scene with an average time of 89 s and a mean precision of 96%. With a crowd of three workers, the segmentation time dropped to an average of 26.5 s, with a decrease in precision of 15%. In Khoo et al. (2015), the authors explore the use of a crowd-based navigation system to assist visually impaired people in navigating public spaces. Utilizing the system, a crowd of 11 participants was able to navigate artificially generated mazes in times ranging from 3 to 4 min. The RoboTurk platform (Mandlekar et al., 2018) employs crowdsourcing to collect robot arm manipulation data for training reinforcement learning models. Relying on contracted workers, the platform was able to collect over 2,200 demonstrations in 20 h of system usage. In Sorokin et al. (2010), the authors relied on the Amazon Mechanical Turk crowdsourcing platform (Amazon, 2005) to segment and annotate 3D scenes into labeled objects, improving robot grasping. Response time and annotation quality on the Turk platform depends on the payment, with rates exceeding 300 annotations per hour for an hourly compensation of 1 USD at the time of the study (Sorokin and Forsyth, 2008).

Although crowdsourcing platforms are an effective way of solving complex reasoning problems through collective thinking, most of them lack crowd motivation, requiring expensive incentives, such as rewards or payments for user participation (Amazon, 2005). An alternative for increasing the participant engagement in problem-solving environments is to provide aesthetically pleasing, easy to learn, intellectually challenging interfaces that entertain and motivate the user, such as gaming platforms. Every year, more than two billion people spend a considerable amount of time daily playing games that test their problem-solving skills in diverse scenarios (Wijman, 2018). The data collected through these robust, synchronized, and high-speed gaming networks can be used to deal with real-world problems, even without players being aware of the process (Cooper et al., 2010; Chernova et al., 2011; Chirayath and Li, 2019). The NASA Neural Multi-Modal Observation and Training Network (NeMO-Net) (Chirayath and Li, 2019) and Foldit (Cooper et al., 2010) are examples of games that extract scientific outcomes and value through player participation. In the first, players identify and classify coral reefs using satellite and drone images, and the data is used to train a Convolutional Neural Network (CNN). The Foldit online game is used to engage non-scientists in predicting complex protein structures.

There are several features that make the combination of gamification and crowdsourcing frameworks specifically applicable to robotic contexts. Robots require sophisticated perception that should be able to sense and understand dynamic and unstructured surroundings to execute tasks with ease (Luo et al., 2017). To accomplish this, the robotic and gaming environments should share common parameters that are based on simulated real-life conditions. In the gaming environment, human players can understand, evaluate, and respond to these simulated conditions by altering their gameplay, improving robot performance (Crick et al., 2011). Such gamification schemes contribute toward a synergistic human-machine collaboration that improves and facilitates robotic problem-solving (Jarrahi, 2018).

Our previous work (Bewley and Liarokapis, 2019), proposes abstract foundations for a framework combining gamification and crowdsourcing in a synergistic manner for robotics applications. The paper also introduces a standardized terminology for describing crowdsourcing techniques in robotics. It discusses some implementation challenges and how gamification can contribute to the cost-effectiveness, privacy, scalability, and ethical integrity considerations of crowdsourcing. However, that work is purely theoretical and does not offer a solution to any specific problem in robotics. Nevertheless, the abstract flow of information in the gamified crowd computer concept served as a guideline in the development of the framework proposed in this work.

This work proposes a crowdsourced attribute matching framework that leverages human perception to support and improve the grasping and manipulation capabilities of autonomous robotic platforms. The system encodes visual information into an engaging online puzzle game, relying on the collective intelligence of players to identify the attributes of unknown objects and images. The game employs a popular tile-matching format, where the players connect images that share the same attributes. Correct matches make the connected tiles disappear, awarding points to the player. A small fraction of unknown images are mixed with known ones, which facilitates attribute identification through the game's matching mechanism. This is used to expand an initial object database and solve perception problems in near real-time. The novel aspect of this work is in the synergistic combination of game mechanics with a crowdsourcing framework for the purpose of enhancing robot perception. The game interface is designed to challenge and entertain the players, as opposed to traditional robotic crowdsourcing approaches, such as Clair et al. (2016) or Kent (2017) that directly expose the robot context and often fail to intrinsically motivate the users to participate without financial compensation. The developed interface also effectively obfuscates the underlying robotic application to address any security and confidentiality concerns. The framework was evaluated in two real-time, proof-of-concept applications: (i) enhancing the control of a wearable robotic exoskeleton glove for assisted manipulation and (ii) improving object identification for autonomous robot grasping. The first was chosen to highlight the framework's capability to operate without a local classifier and demonstrate its suitability for applications in remote, assistive robotics. The second was chosen to validate its performance in a more industrial vision task, in synergy with a dedicated local classifier.

## 2. Framework Design

**Figure 1:**
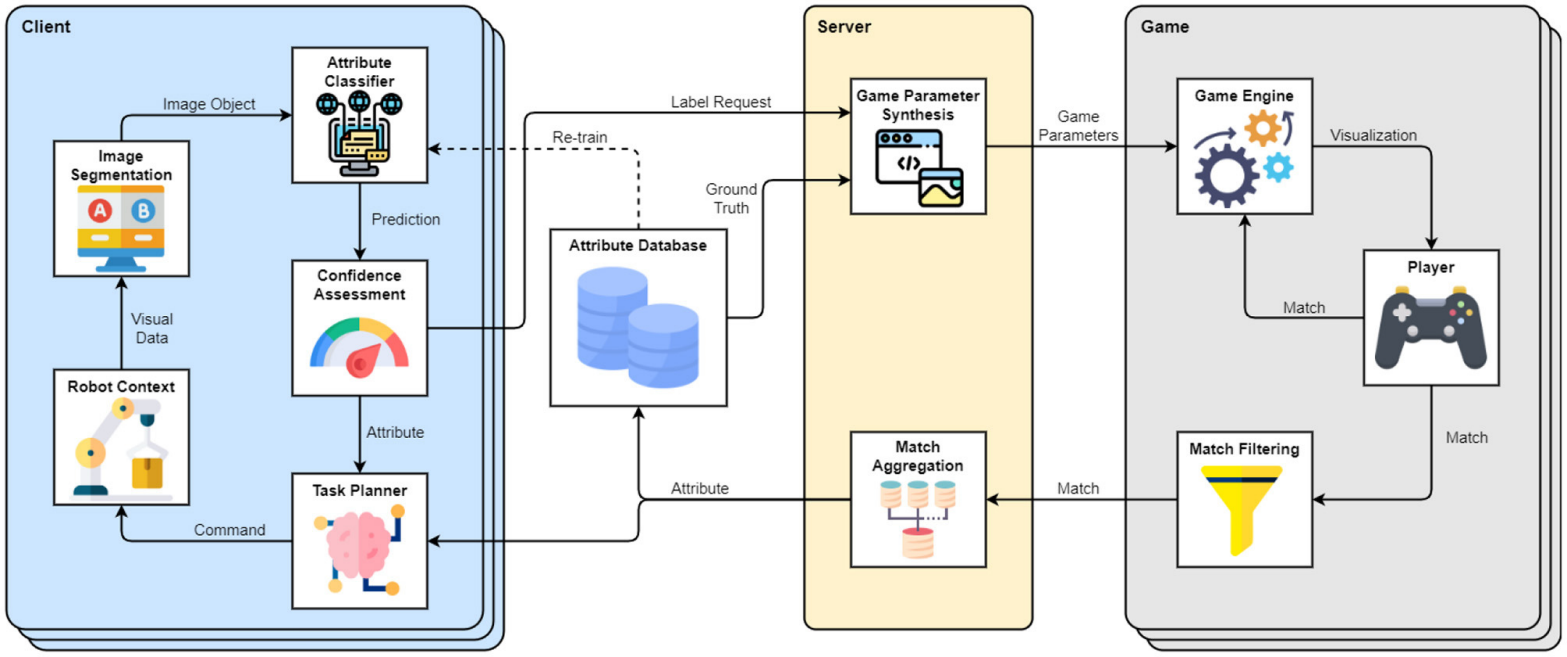
Structure and flow of information within the proposed attribute matching framework. The example client manages a Robot Context that relies on an Attribute Classifier to characterize objects detected in the robot environment. Predictions of the Attribute Classifier are assessed based on their confidence, and the client can request assistance with labeling the low-confidence predictions. The Label Request passes to the server, which synthesizes Game Parameters by combining ground truth and received data. The Game Parameters are passed to the Game Engine, which is accessible to the users. The players create matches between the known and unknown images based on their attributes. The matches are filtered and returned to the server, which aggregates them to estimate the Attributes of the unknown images. The results are passed to the client, which can use them directly in the Task Planner. When a label reaches high enough confidence and number of matches, it becomes known / validated and is included in the database, which can be utilized to re-train the classifier of the client. The framework is flexible on the client side and can be adapted to arbitrary task specifications. Icons were sourced and modified from Flaticon (2010).

**Figure 2:**
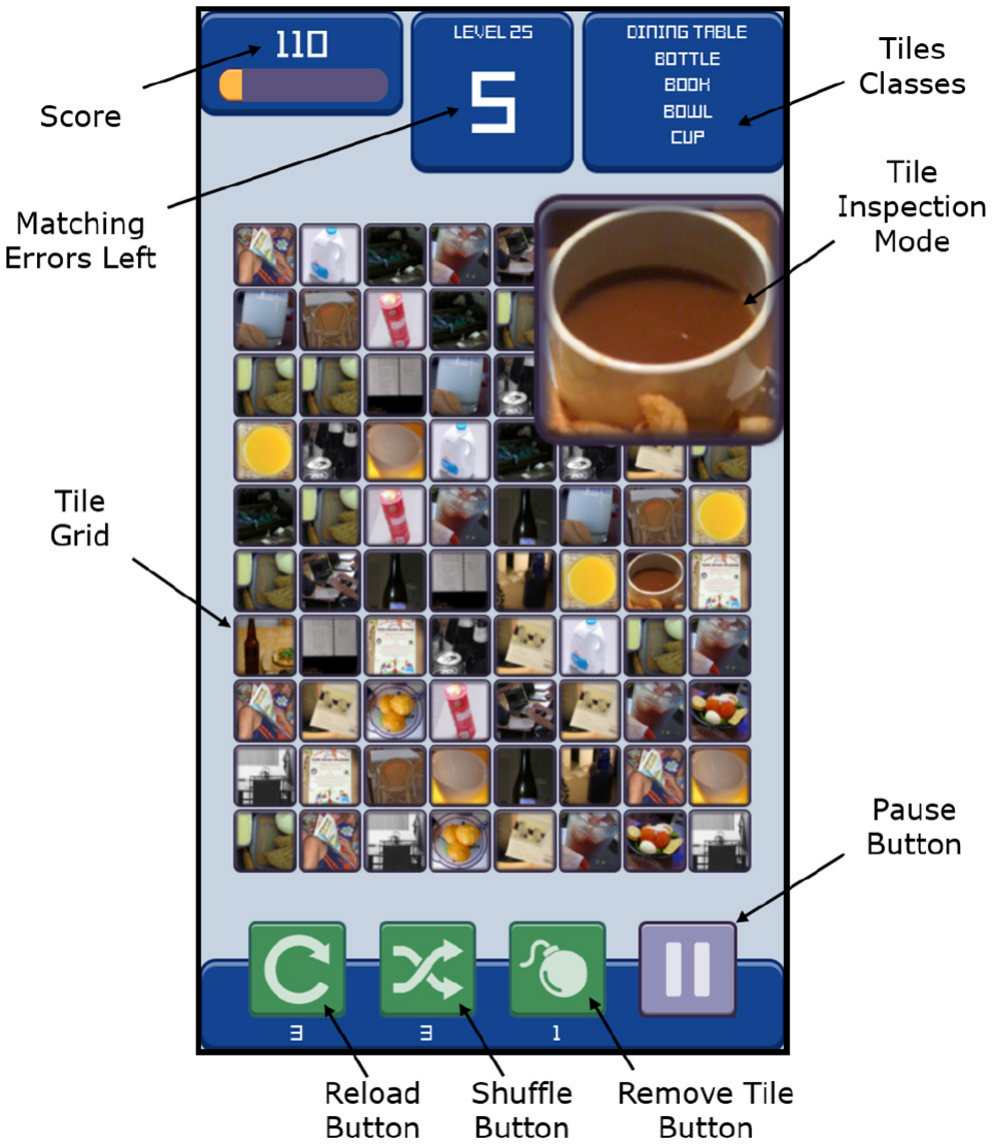
Interface of the developed tile-matching game. The player matches adjacent tiles based on the classes listed in the top right corner. To facilitate attribute recognition of individual tiles, they can be enlarged using the tile inspection mode. If connected tiles share the same attribute, they disappear and increase the player score. The number of permitted matching errors per level is limited, which encourages the player to inspect the tiles closely before connecting them. If players are stuck on a level, they can utilize boosters that can either reload the board, shuffle the tiles, or remove a specific type of tile. The game is compatible with Windows, Linux, Android, iOS, and HTML5 to allow running in a web browser.

A high-level diagram of the framework interacting with a group of clients is depicted in Figure 1. In this setting, the framework consists of three modules: a client managing a robot context, the server handling client requests and generating game parameters, and a game that is distributed to players for crowdsourcing. A client can also be a group of clients. Each client is solving a task that requires the extraction of characteristics of objects that exist in the robot's environment and are captured through a vision-based system. Typically, the client accomplishes that using a Machine Learning method (marked as Attribute Classifier in Figure 1), which processes segmented scene images and outputs attribute predictions with certain confidence values. If the prediction confidence is sufficiently high for a particular object, the client needs no assistance and execution can continue autonomously. However, when encountering predictions with low confidence or objects that the classifier was not trained on, the Confidence Assessment module may submit a Label Request to the framework and rely on the players to obtain an estimate of the unknown object attribute. The Label Request contains an image of the object in the scene, as well as its attribute group that describes what kind of attributes the players should look for (e.g., stiffness and object class). The Server collects label requests from multiple clients and uses them to construct parameter sets for game instances distributed to the players. The game parameters include approved labels from the Attribute Database, as well as a small fraction of unknown images sampled from the label requests. The Game Engine encodes the received images into the developed tile-matching game presented in Figure 2. In the game, players receive points for matching three or more tiles that share the same attributes. The web interface offers a leveling system that unlocks in-game rewards, as well as a leaderboard that increases competitiveness and motivation. The players are incentivized to enlarge and closely inspect the tiles before matching, as the level is lost after a number of matching errors. Every time an unlabeled tile is paired with two or more tiles of the same type, the match is sent to the server. The server aggregates matches received from all active game instances and updates the status of labels submitted by the clients. Once a sufficiently large number of matches for a specific label is reached and the crowd consensus exceeds a chosen confidence threshold, the label is approved/validated and is included in the database for future use. Once the label is approved, the new information (Attribute) is passed to the Task Planner for immediate use in generating robot commands. The framework and an online version of the game are deployed and available at: https://www.newdexterity.org/aispy.

The framework is very flexible on the client side and does not impose a specific structure on their solution design. Depending on the task specifications, the clients can choose to periodically re-train their Attribute Classifier on the growing database to improve performance over time. In case re-training becomes computationally too expensive as the database grows in size, clients may use only a limited subset of labels to train on. They also have full control over the images they submit, and they are free to delete any of their own labels to keep their database size under control. Alternatively, the classifier can be automatically trained in a continuous manner through appropriate reinforcement learning methods (Gu et al., 2017; Johannink et al., 2019) to reduce the amount of human involvement. Apart from its effects on model training, the knowledge acquired over time does not negatively impact the framework performance and is limited only by the amount of available server storage. The framework can only assign pre-defined attribute classes to new images, which means that the initial client configuration does not get changed through crowd participation. Clients are also free to choose how much trust they place in the prediction of their classifier. They can use the proposed framework to verify only predictions with lower confidence, or they can choose to employ both the framework and classifier for every prediction, in order to establish an additional layer of verification. This behavior may be controlled through thresholds in the Confidence Assessment module.

### 2.1. Server Architecture

The server accepts label requests from clients, encodes them into game instances, and interprets the results. It consists of three main components: the attribute database, the client Application Programming Interface (API), and the game engine API. In addition, it offers a simple website for user registration and access to the game.

For storing the user, image, and label data, a relational database type is employed. Registered users can act as clients or players, opting in to link their identity to any images, label requests, or matches submitted to the server. The database can be initialized with an arbitrary number of image collections and ground truth labels. Each label contains an image reference, bounding box, attribute group, attribute name, and confidence value. The attribute group represents a high-level description of object attributes, such as “stiffness” or “object class.” The matches submitted by players are stored in a separate table and linked to their respective label requests. Once the number of matches for a particular label exceeds the set threshold, the most common match is assigned as label attribute and the label confidence is calculated as the ratio between the number of most common matches and the total number of matches. A label request is accepted as ground truth when the number of matches reaches a certain threshold and its confidence value exceeds 95%. These values can be adjusted according to the client and task specifications.

On the client side, the server allows for label submission and querying through the Hypertext Transfer Protocol (HTTP). The clients are able to submit label requests paired with new images, or in reference to existing images stored on the server. After submission, clients can receive updates for their label requests in terms of confidence and number of matches. Clients may also withdraw any labels they submitted. On the game side, the server offers an HTTP API for requesting tile textures and submitting matches with unlabeled tiles. Upon request from a game instance, the server randomly selects one or more active label requests, along with a number of ground truth labels from the same attribute group. These are compiled into an atlas image and sent to the game instance, along with the attribute names of ground truth labels, and the identities (ID) of the label requests. When a valid match that includes an unknown image is submitted by the player, the game sends an update containing the label ID and attribute name back to the server.

### 2.2. Game Interface

The game design was inspired by the addictive tile-matching genre of video games, where players manipulate tiles in order to make them disappear according to a defined matching criterion. In the game, the player is presented with an 8 × 10 grid of tiles overlaid with the images received from the server, as depicted in Figure 2. Each tile is tagged with its corresponding attribute name if the image is known, or a label request ID if the image is unknown. The player can highlight a chain of three or more adjacent tiles which disappear if their attributes match. A match is also accepted if the tile chain includes known tiles with the same attribute and a single unlabeled tile. In this case, the game submits to the server a match that links the unlabeled tile with the common attribute of the rest of the chain. To authenticate their matches, users are required to input their credentials before starting the game. For completing a level, the player must reach a target score by performing successful matches. The number of permitted matching errors per level is limited, which motivates the players to inspect the tiles closely before creating a match. To facilitate this, the player can enlarge any tile in inspection mode, which is particularly valuable for mobile devices. To help players identify matching tiles, the list of relevant attributes is displayed in the top right corner of the game interface. The players are also motivated through a leveling system and an online leaderboard. Upon leveling up, helpful boosters, such as “shuffle” and “reload” are unlocked to prevent players from getting stuck. Early levels in the game are configured to contain only known tiles in order to train new players and familiarize them with the game mechanics. Higher levels require a higher score to complete, and permit a lower number of matching errors. The game can be exported for Windows, Linux, Android, iOS, or HTML5 to run in a web browser.

### 2.3. Security

In order to ensure responsive gameplay, the game runs fully on the user's device. This presents a security risk, since individuals with malicious intent might abuse the game-server communication to submit artificial matches and sabotage the labeling system. To prevent this, all critical communication with the server, such as matching and level completion, must be accompanied by what we call a Proof of Play. The server provides each game instance with a random seed that is used to populate and refill the tile grid. Proof of Play includes this seed, and the sequence of player actions leading to the current game state. Before accepting any request from a game, the server can therefore check the player's actions and verify that the game was actually played. This drastically reduces the risk of system exploitation and provides added security to the client applications.

## 3. Matching Rate Estimation

Performance of the framework from a client's perspective can be characterized through the time required to resolve a submitted label request. Assuming a constant matching rate of individual players, the expected number of matches per submitted label *m* over time *t* can be estimated by:

(1) $$\begin{array}{r} m=\frac{c_{m}\cdot p\cdot a_{g}}{d\cdot l\cdot a_{t}}\cdot t, \end{array}$$

where *c*_*m*_ is the matching constant, *p* is the number of active players, *a*_*g*_ is the number of attributes sampled in each game instance, *a*_*t*_ is the total number of attributes in the attribute group, *l* is the number of active labels, and *d* is the matching difficulty. The matching constant *c*_*m*_ is static for a given game format, and can be estimated from live game data by monitoring and/or varying the other parameters. The role of *p* and *l* is intuitive; a higher number of players increases the matching rate, while a higher number of candidate labels decreases it. The ratio between *a*_*g*_ and *a*_*t*_ represents the matching capacity; if a given attribute group contains more attributes than a single game instance can contain, there is only a *a*_*g*_/*a*_*t*_ chance that the unknown label can be matched with its true class. In other words, a randomly sampled attribute group with dozens of classes will produce only a handful of cases in which the unlabeled images are mixed with known images that share the same attribute. Finally, the matching difficulty *d* is defined in terms of similarity between objects (images) with different attributes. In this setting, the measure of similarity was based on object super-categories of the COCO dataset (Lin et al., 2014). For instance, matching between images that all belong to a single super-category (e.g., fork, knife, spoon) is more difficult than matching between diverse super-categories (e.g., orange, tv, teddy bear). The matching difficulty was therefore defined as:

(2) $$\begin{array}{r} d=\frac{a_{g}}{\text{E}\left[s_{g}\right]}, \end{array}$$

where E[*s*_*g*_] represents the expected number of different super-categories in a game instance *s*_*g*_. This is obtained by:

(3) $$\begin{array}{r} E\left[s_{g}\right]=\overset{a_{g}}{\underset{i=1}{\sum }}i\cdot \text{P}\left(s_{g}=i\;|\;a_{g}\right), \end{array}$$

where P(*s*_*g*_ = *i* | *a*_*g*_) represents the probability of sampling *i* super-categories in a game instance, given the sample size of *a*_*g*_. This can be calculated as the ratio between possible samples that contain *i* super-categories and all possible sampling combinations:

(4) $$\text{P}\left(s_{g}=i\;|\;a_{g}\right)=\frac{\left[x^{a_{g}}y^{i}\right]G(x,y)}{\left(\begin{array}{c} a_{t}\\ a_{g} \end{array}\right)}.$$

Here, *G*(*x, y*) is a bivariate generating function of the form $G\left(x,y\right)=\underset{m,n\geq 0}{\sum }g_{m,n}x^{m}y^{n}$, while [*x*^*m*^*y*^*n*^]*G*(*x, y*) refers to coefficient *g*_*m, n*_ of *G*(*x, y*). The expression atag represents all possible combinations of sampling *a*_*g*_ attributes from a total of *a*_*t*_, computed through a binomial coefficient. The generating function *G*(*x, y*) is constructed to have the *x* variable tracking the number of sampled attributes, and *y* tracking the number of super-categories:

(5) $$G(x,y)=\prod_{i=1}^{\left|S\right|}\left(1+\sum_{j=1}^{s_{i}}\left(\begin{array}{c} s_{i}\\ j \end{array}\right)\;\cdot \;x^{j}y\right),$$

where *S* is the set of all super-categories in the attribute group, with |*S*| ≤ *a*_*t*_, and *s*_*i*_ is the number of attributes belonging to the *i*-th super-category of set *S*.

## 4. Evaluation Methods

This section presents the experimental setup and methods for evaluating the attribute matching performance of the proposed framework, as well as the apparatus and methods for the two proof-of-concept applications.

### 4.1. Attribute Matching

In the first stage of experiments, the attribute matching capability of the proposed framework was evaluated in terms of labeling accuracy and matching rate. For this purpose, the system was deployed on a local network, accessible to a subject group of 25 players. After registering with the game server, the players were given a brief introduction to the game mechanics, as well as some time to practice. The game was configured to receive five random attribute classes with five different images per class, plus an additional two unlabeled validation images. The chosen attribute group for the evaluation experiments was “object type,” which corresponds to a traditional object classification problem. Object images were sourced from the COCO database (Lin et al., 2014), in particular from the 2017 training set. The experimental evaluation consisted of multiple runs, where the seed database of the framework was initialized with 5, 10, and 15 randomly selected attribute classes (object types). In each run, a group of 2 and 5 validation images were submitted to the framework for labeling. For each validation image set, the number of matches and the label confidence for each image was monitored over a time period of 10 min, with 25 users playing simultaneously. The recorded data was also used to estimate the matching constant *c*_*m*_.

### 4.2. Classification Confidence Interval Estimation

In order to evaluate the proposed framework, the results obtained during the object classification experiments with the robotic exoskeleton glove and the intelligent robotic platform require an estimation of their classification confidence. The confidence intervals for binary classification can be estimated through a number of methods, where the simplest and most common approach relies on approximating the error with a standard normal distribution. However, this approach has been shown to perform poorly, especially with small sample sizes and in cases where the expected success proportions are close to 0 or 1 (Brown et al., 2001). Since some samples obtained through the framework are likely to fall into the above categories, the confidence intervals are estimated through the method introduced by Wilson (1927), which offers better performance in corner cases. For proportion *p*, the Wilson confidence interval is defined as follows:

(6) $$\begin{array}{r} w^{-}<p<w^{+} \end{array}$$

The lower limit *w*^−^ and upper limit *w*^+^ of the interval for confidence α can be obtained through:

(7) $$\begin{array}{r} w^{-},w^{+}=\frac{1}{1+\frac{z_{\alpha /2}^{2}}{n}}\left(\hat{p}+\frac{z_{\alpha /2}^{2}}{2n}\right)\pm \frac{1}{1+\frac{z_{\alpha /2}^{2}}{n}}\sqrt{\frac{\hat{p}\left(1-\hat{p}\right)}{n}+\frac{z_{\alpha /2}^{2}}{4n^{2}}} \end{array}$$

Where $\hat{p}$ is the observed success proportion, *n* is the number of matches, and *z*_α/2_ is the z-score for which the area of α/2 is found under the normal curve. The classification interval calculation for a high level of confidence (95%) gives an estimate of the reliability of the framework results for a group of players.

### 4.3. Enhancing the Control of a Wearable Robotic Exoskeleton Glove

**Figure 3:**
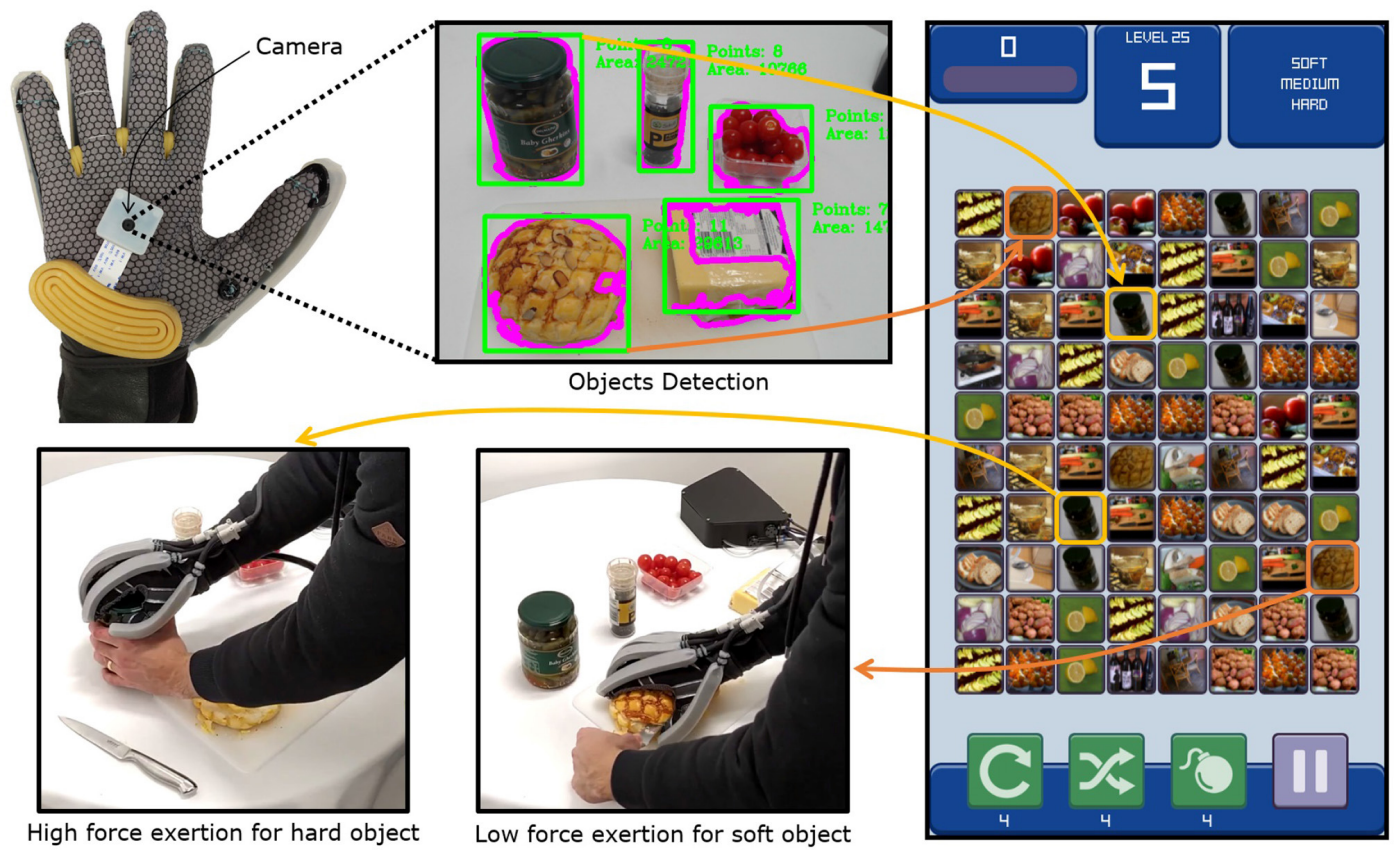
Information flow in the exoskeleton glove control enhancement experiment. The camera mounted on the exoskeleton glove captures the scene image, where object bounding boxes are detected through edge detection and contour extraction. The unknown object images are submitted for labeling to the server, within the “stiffness” attribute group. The server generates game instances where the players connect objects that share the same stiffness attribute (“soft,” “medium,” “hard”). Player matches are aggregated and the assigned stiffness attributes of unknown objects are returned to the glove. As the glove gets pre-positioned above an object, the object image is linked to the labeled results by comparing its ORB (Rublee et al., 2011) descriptors. When the user attempts to grasp an object and occludes the palm camera, the glove triggers a grasp that exerts an appropriate amount of force on the object, with respect to its stiffness.

The first application focused on utilizing the framework for enhancing the control of a wearable, soft robotic exoskeleton glove for assisted manipulation in a food preparation task. In the experiment, the exoskeleton glove developed by the New Dexterity research group was used (shown in Figure 3). The exoskeleton glove was designed to assist human hands with limited mobility during the motion rehabilitation process and to improve the grasping and dexterous manipulation capabilities of the hand, in both impaired and able-bodied individuals. The device is composed of a glove, a tendon-driven system with six tendons (five tendons for finger flexion and one for thumb opposition), and a pneumatic system that consists of four soft actuators and five laminar jamming structures. More details regarding the exoskeleton glove design and operation can be found in Gerez et al. (2020). A small camera (Raspberry Pi Camera Module V2, Raspberry Pi Foundation, UK) was mounted on the palm of the robotic exoskeleton glove to capture images for the object tracking algorithm during task execution.

The kitchen is one of the most complex environments for robots in terms of control complexity when attempting to grasp and manipulate objects. The variety of object shapes, textures, and materials make such robot-assisted tasks still a challenge for the current exoskeleton glove devices (Zhou and Ben-Tzvi, 2014; Chu and Patterson, 2018). For this reason, this experiment consisted of executing force-controlled, cooking-related tasks with the assistance of the exoskeleton glove. A ground truth database of 100 common kitchen object images was constructed and labeled to initialize the server. The images were labeled as “soft,” “medium,” or “hard” within the “stiffness” attribute group. Since the seed database was very small, it was not possible to train an attribute classifier that relies only on traditional Machine Learning methods in order to reliably estimate the stiffness of completely new objects. Such issues with training set size are common in robotic applications, reinforcing the need for solutions based on gamification and crowdsourcing.

In the experiment, a set of common kitchen objects was placed on a table and the user was equipped with the exoskeleton glove. Initially, the glove was pointed to the table so that the camera could capture all the objects in its field of view. The object bounding boxes were segmented from the video frames by first applying the Canny edge detector (Canny, 1986), dilating the result, extracting closed contours (Suzuki et al., 1985) and finding their bounding rectangles. Since no trained attribute classifier was available for this task, all extracted objects were sent to the game server for labeling. The participants who played the game thus received the unknown object images from the glove environment as additional tiles in the game, which they connected with the ground-truth tiles to identify their stiffness. The glove wearer waited until all submitted label requests for unknown object stiffness were classified with at least three matches. As the glove moved across the scene, the bounding box closest to the center of the video frame was considered to be the target object for grasping. To find which object corresponds to the central bounding box, ORB keypoints and descriptors (Rublee et al., 2011) were extracted from the central bounding box and the initial images labeled by the framework. The central bounding box in the live feed was thus linked to the object with the highest number of descriptor matches. When the wearer attempted to grasp an object by touching it with the palm, the camera view got occluded, shading the image. The relative image brightness was thus used to trigger the grasp. With the stiffness of the grasped object known, the glove was able to apply the ideal amount of force to successfully execute the task. The maximum force applied by the robotic exoskeleton glove was set for each level of stiffness (soft, medium, and hard) by limiting the maximum current applied to the motors of the tendon-driven system (which assists on the execution of the fingers flexion). The players were not aware which underlying perception problem they were solving through gameplay, which demonstrated the generalization and obfuscation capabilities of the proposed framework. A total of five objects were labeled in this experiment, with 25 users playing the game simultaneously. The matching rates for individual objects were also compared with predicted values over a time period of 10 min.

### 4.4. Improving Object Identification for Autonomous Robot Grasping

**Figure 4:**
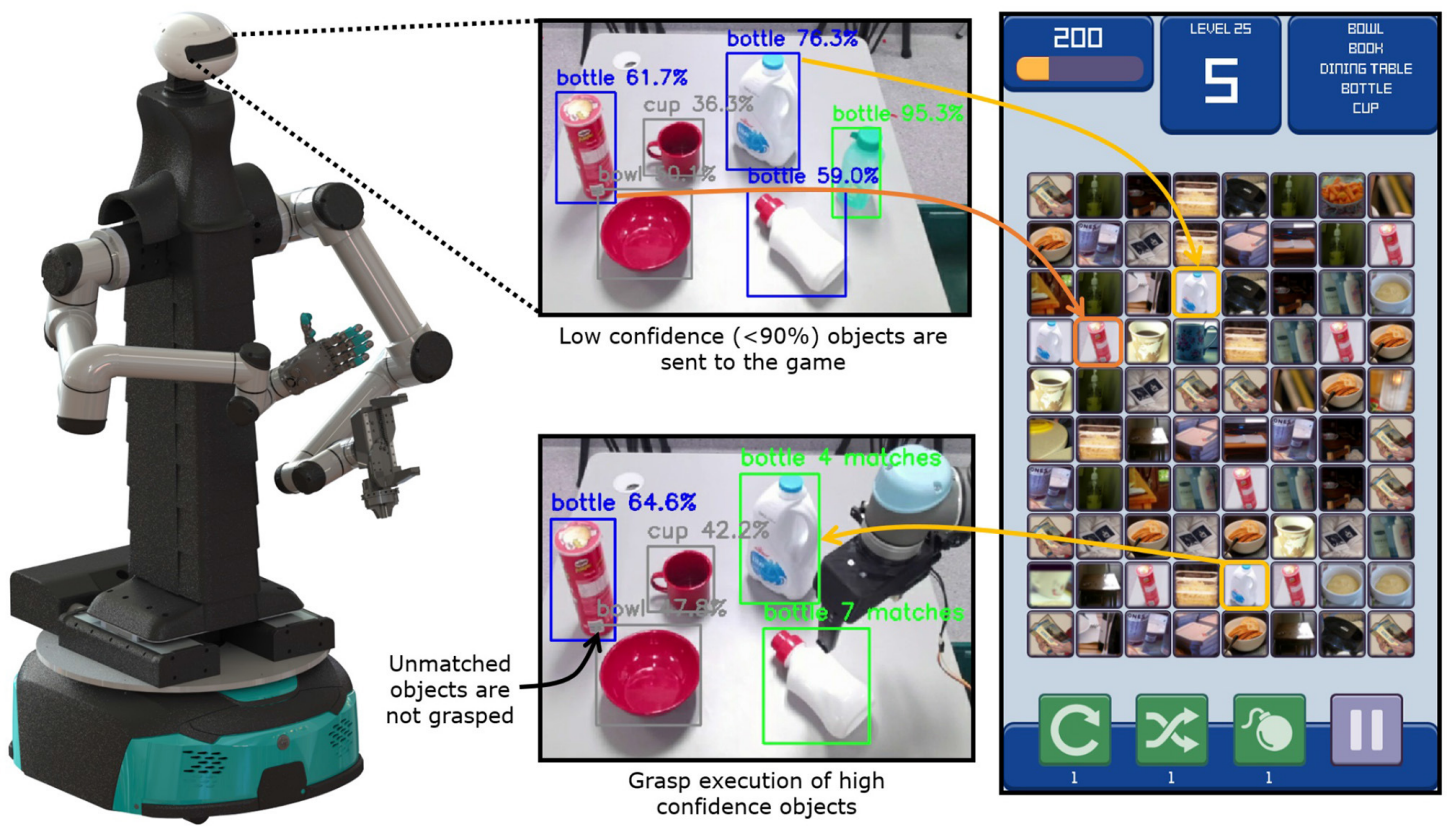
Information flow in the object identification for the autonomous robotic grasping experiment. The camera in the robot head captures the scene image, which is processed by a pre-trained Convolutional Neural Network (CNN) (Huang et al., 2017). The network detects and classifies the objects in the robot scene, highlighting in green the bottles with high confidence, and in blue bottles with lower confidence. The bottles with high confidence are immediately picked up and disposed of, while bottles with low confidence are submitted to the framework for identification. The server generates game instances where the players connect objects that share the same class (bottles, cups, bowls, etc.). The player matches are aggregated and the assigned classes of unknown objects are returned to the robot. As the confidence of detected bottles gets refined by the framework, they are scheduled for pick-up. The chips can is incorrectly classified by the CNN, but the players reject the classifier prediction, preventing a sorting error.

In the second application, the developed framework was employed for executing autonomous robotic grasping tasks. In particular, it refined the perception estimates of a trained object detection and classification algorithm. The task consisted of detecting and picking out bottles from a group of objects with an intelligent robotic platform developed by the New Dexterity research group at the University of Auckland, as shown in Figure 4. The platform is equipped with two 6-DoF serial manipulators (UR5, Universal Robots, Denmark), a reconfigurable torso, and a head that acts as the perception system. Only one arm was used in the experiments, equipped with an adaptive gripper also developed by New Dexterity (Gorjup et al., 2020). The gripper consists of a 3-fingered rotary module and a parallel-jaw element. The 3-fingered rotary module utilizes a scroll wheel mechanism and a clutch to perform grasping and rotational motions. The parallel-jaw element uses a rack and pinion mechanism to execute grasping motions with a pair of fingers with compliant finger pads. The head module of the robot houses an Azure Kinect DK module (Microsoft Corporation, USA), which streams RGB and depth data of the observed scene.

In the experiment, six objects were placed on a table surface in front of the bimanual robotic platform, as depicted in Figure 4. The objects consisted of a bowl, a cup, a chips can, and three bottles of different sizes and shapes. The RGB video stream of the scene was processed by a CNN, pre-trained on the COCO database (Huang et al., 2017). The network produced results in terms of object classes, confidences, and bounding boxes that were drawn on the output image for visualization. To enable the generation of 6D grasp poses, object clusters were segmented from the depth cloud data stream. Object centroids and principal axes were computed by applying the dimensionality reduction method Principal Component Analysis (PCA) (Artac et al., 2002) to the object point clusters. The grasp position was selected as the cluster centroid, while the grasp orientation was computed with respect to the cluster's principal axis. The object clusters were connected to their bounding boxes by projecting their centroids to the RGB image and matching them based on the distance from the centers of the bounding boxes.

The network outputs were filtered to highlight the bounding boxes of any detected bottles in the scene and depict other classes in gray. Any bottles that were detected with confidence higher than 90% by the CNN were immediately scheduled for pick-up. Predictions with lower confidence were submitted for re-evaluation to the game server while the robot picked up the high-confidence bottles. This allowed for a parallel execution of the perception processing tasks in a synergistic manner between the humans and the robot. Any lower-confidence objects that were labeled as bottles by the game server were scheduled for pick-up by the robot. Labeling was performed with 25 users playing the game simultaneously. The labeling time and confidence were recorded and the matching rates were again compared with the theoretical values.

## 5. Results

### 5.1. Attribute Matching

**Figure 5:**
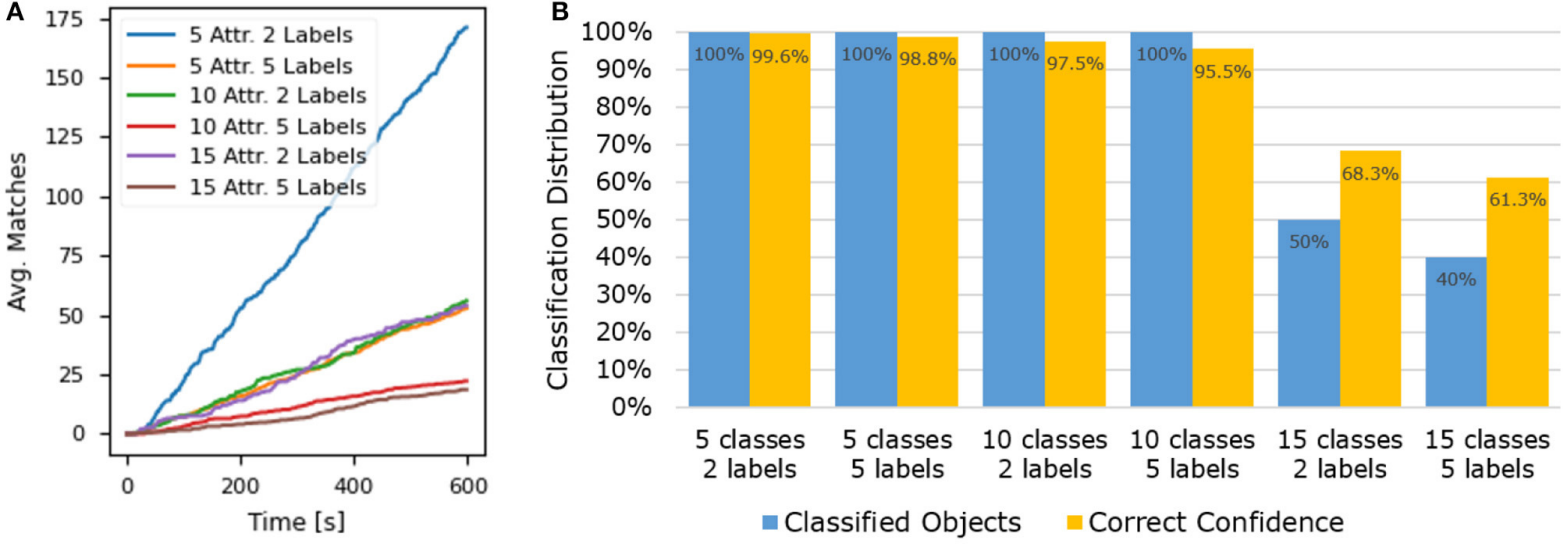
Results from the framework evaluation experiments. **(A)** Presents the average number of matches per validation label over time, with respect to the number of attribute classes and the number of submitted validation labels. **(B)** Presents the classification distribution and confidence for the evaluated test cases with varying numbers of attribute classes and unknown validation labels. A label was considered classified if it reached 5 or more player matches, a confidence exceeding 70%, and the assigned crowd attribute was correct. The correct confidence was computed as the ratio between the correct and total number of matches.

The attribute estimation accuracy and response time of the proposed framework were evaluated through a series of experiments where small sets of known images were submitted for processing. Performance was assessed under different conditions, varying the size of the validation image set, and the number of different classes in the attribute group. Figure 5 displays the framework evaluation results for a group of 25 players in six different conditions (attribute groups of size 5, 10, and 15, with 2 and 5 validation labels). In Figure 5A, it is visible that the matching rate is linear, as assumed in the model proposed in section 3. The effects of the attribute group size and of the submitted label density on matching speed can also be observed. The recorded data was used to estimate the matching constant *c*_*m*_, with respect to the proposed linear model. The matching constant was computed for each test case, resulting in the average $c_{m}=0.0241\;{\text{s}}^{-1}$, with a standard deviation of 0.00408 s^−1^. Figure 5B shows that the proposed framework performs best when the number of ground truth attribute classes is low. With attribute group sizes of 5 and 10, all validation labels were correctly classified by the system, with a confidence of well over 90%. In the last case of 15 attribute classes, the percentage of classified labels and the average confidence are considerably lower, which is mostly due to the lower matching rate and higher chance of misclassification. This can be mitigated by employing a larger number of players or by including label hints that limit the permissible number of attribute classes in the game. Larger attribute groups can also be divided through clustering methods to boost the labeling performance. Overall, the results demonstrate that the framework can be efficiently combined with robotic systems that are able to provide a set of initial guesses with the submitted label requests, limiting the permissible number of attribute classes in the game.

### 5.2. Enhancing the Control of a Robotic Exoskeleton Glove

**Figure 6:**
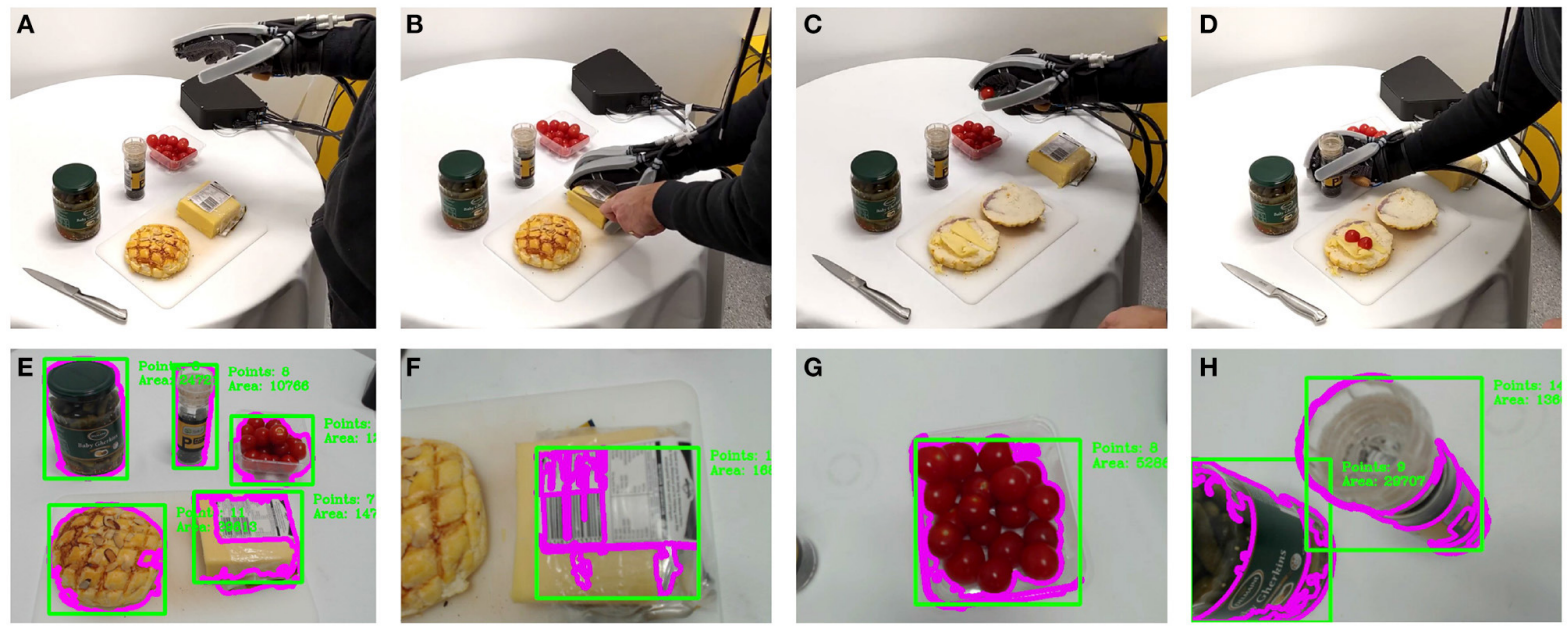
Instances from the execution of the exoskeleton glove control enhancement experiment. The top row shows the robotic exoskeleton glove executing the experiment. The bottom row shows the camera field of view **(E)** with the detected objects. **(A)** Presents the user capturing a scene image that is segmented and submitted for labeling to the framework. **(B–D)** Present the user grasping examples of medium (cheese), soft (tomato), and hard (pepper) objects. **(F–H)** Present the camera view during reach to grasp motions for the cheese, tomato, and pepper objects, respectively.

The first set of experiments focused on validating the proposed framework in estimating the stiffness of multiple objects during the execution of cooking tasks using a robotic exoskeleton glove. The goal of these experiments was to intuitively assist the user in performing manipulation tasks, without them controlling the amount of force necessary to grasp the objects, while the players were asked to match objects with similar level of stiffness in the game (soft, medium, or hard). Figure 6 depicts the four critical steps involved in the task execution: subfigure (A) shows the soft robotic exoskeleton hovering over the scene to detect objects, subfigure (B) shows the user cutting a slice of cheese, while the exoskeleton glove grasps the block of cheese with medium force (for “medium” object stiffness), subfigure (C) presents the exoskeleton glove grasping a cherry tomato with low force (for “soft” object stiffness), subfigure (D) shows the exoskeleton glove grasping a black pepper grinder with high force (for “hard” object stiffness), subfigure (E) depicts the camera view of the scene and the objects detected, and finally, subfigures (F–H) present the camera field of view for the objects detected (cheese, tomato, and pepper grinder) before they were grasped. The bounding box closest to the center of the video frame was considered to be the target object for grasping.

**Figure 7:**
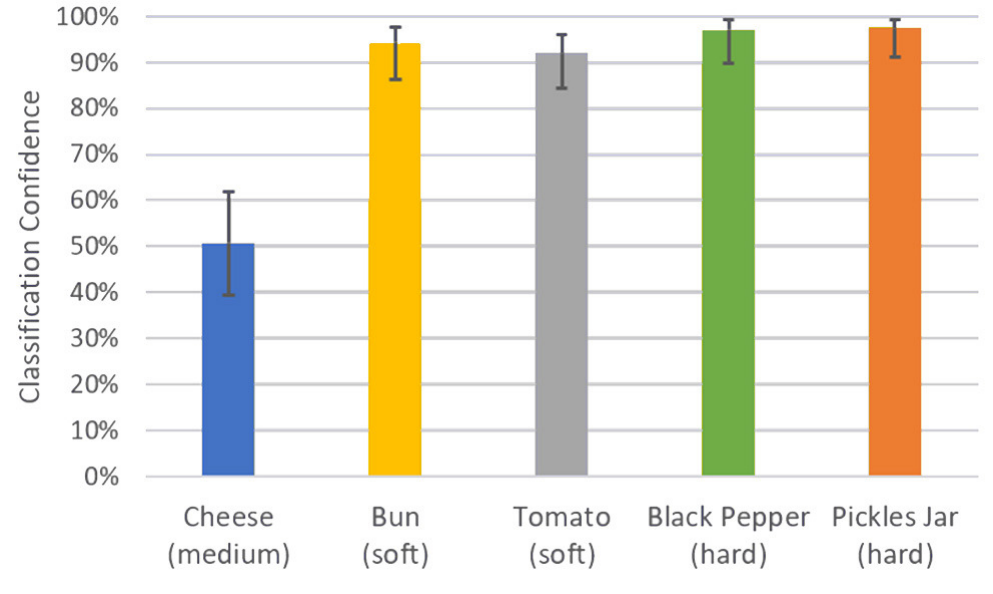
Average classification confidence for the exoskeleton glove control enhancement experiment. The total number of matches over a period of 10 min was 375, with 25 players playing the game simultaneously. The average confidence interval for the object stiffness classification experiment was 82.1% < *p* < 89.1%.

**Figure 8:**
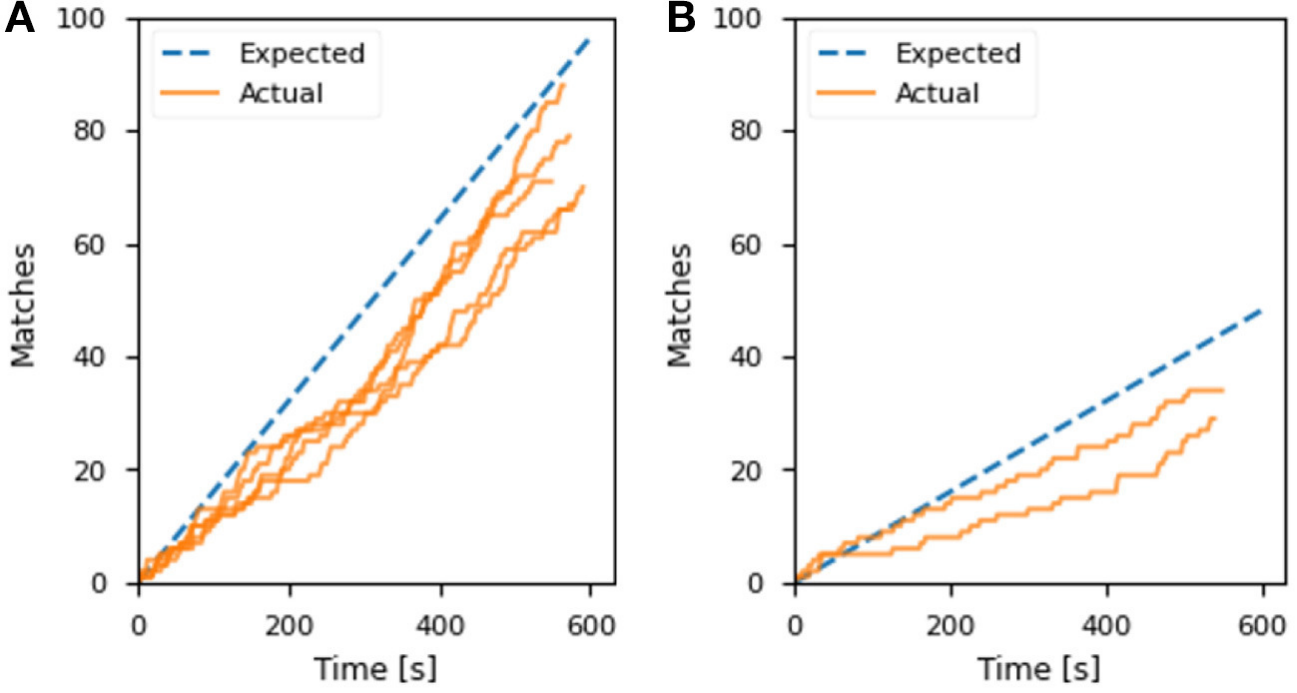
The predicted and actual number of matches over time in the **(A)** control enhancement of the exoskeleton glove experiment and **(B)** object identification for autonomous grasping. The time is counted from the first received player match.

Figure 7 shows the classification confidence and interval in terms of stiffness for each object during the experiments. All objects except the cheese were successfully classified with a confidence above 90%. The lower confidence obtained for the block of cheese can be associated with the lack of consensus on which level of stiffness the object belongs to. The comparison between the actual and predicted matching rate for the exoskeleton glove experiment is presented in Figure 8A. It is visible that the actual values closely match the prediction, which was computed with respect to the matching constant *c*_*m*_ estimated in section 5.1. Although the stiffness identification process was not instantaneous, it could theoretically be reduced to under 1 s if approximately a thousand players were playing the game at the same time (which represents <0.01% of the number of users actively playing games in the Steam platform alone Steam, 2020). Conversely, the glove would perform poorly with fewer players, since it does not have a dedicated classifier to fall back on. To avoid such issues, robot systems should employ a local classifier whenever possible, and rely on the crowdsourcing framework to verify its predictions. Overall, the experiments demonstrate that the combination of the gamification framework and the on-board camera of the exoskeleton glove can assist the user by controlling the grasping forces completely autonomously and in successfully executing a series of tasks in unstructured environments.

### 5.3. Improving Object Identification for Autonomous Robot Grasping

**Figure 9:**
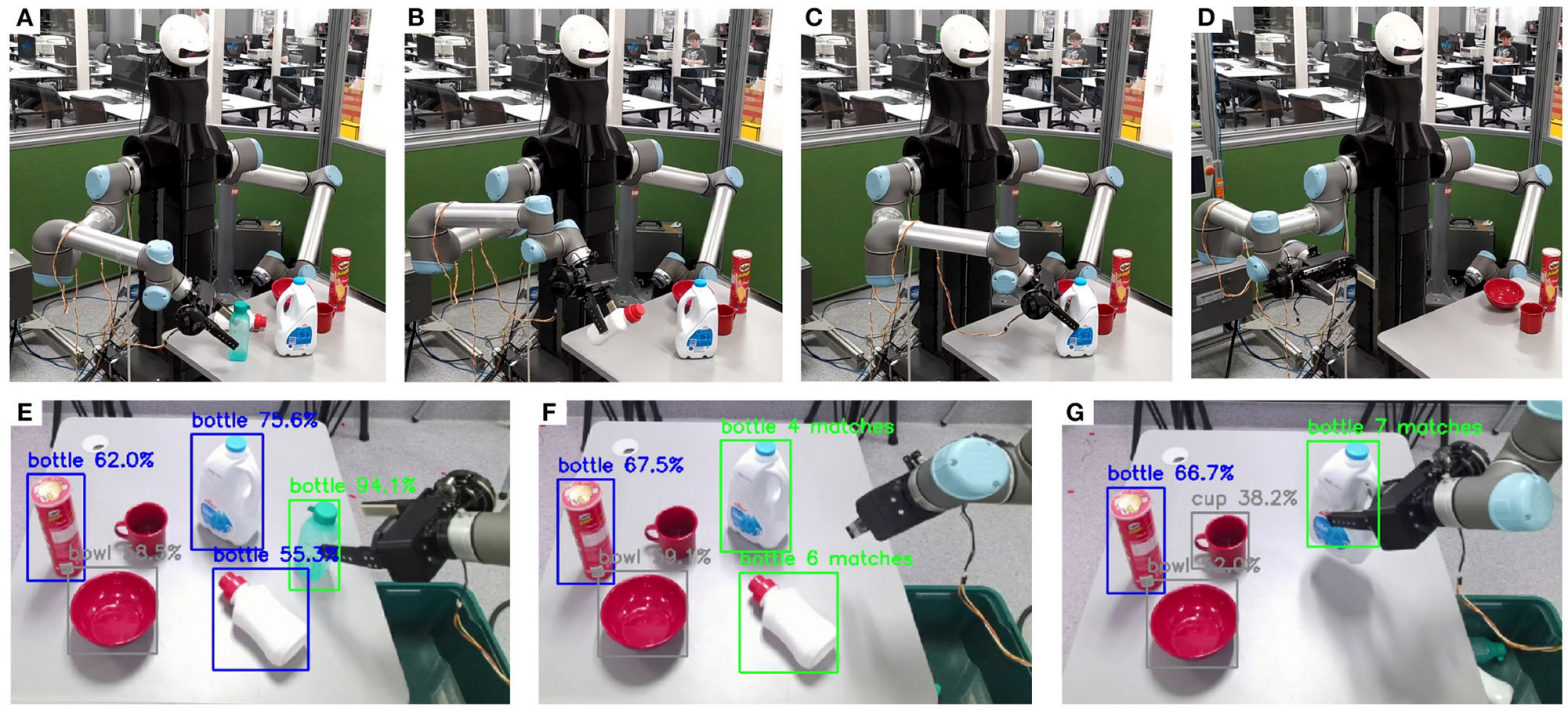
Instances from the execution of the object identification experiment that facilitates autonomous robot grasping. The top row shows the robotic platform executing the experiment. The bottom row shows the camera field of view with the detected objects. **(A–C)** Present the robotic platform grasping and disposing bottles placed arbitrarily on a table, while **(D)** presents the completed bottle sorting task. **(E)** Presents the camera view during grasping of the bottle that was identified with high confidence by the CNN. **(F,G)** Present the camera view during grasping of bottles that were identified with low confidence by the CNN, but confirmed through the proposed attribute matching framework by the players.

The second set of experiments focused on evaluating the proposed framework in improving object identification for grasping and sorting objects using an autonomous robotic system. The goal of the experiment was to collect user data through the gaming platform to optimize object identification for bottle sorting, improving the classification confidence of a pre-trained CNN. Figure 9 presents the different critical stages involved in the task execution: subfigures (A–C) present the intelligent robotic platform grasping and disposing bottles that are arbitrarily positioned in the environment, while subfigure (D) presents the completed bottle sorting task. Subfigure (E) shows the initial confidence values for the objects in the scene (bottles detected with confidence higher than 90% by the CNN were scheduled for pick-up), subfigure (F) presents the objects identified as bottles which were submitted for re-evaluation to the game server, receiving at least three matches, while, finally, subfigure (G) shows the sorting of bottles identified by the game, leaving on the table the cup, the bowl, and the chips can. The chips can was misclassified by the CNN as a bottle, but this prediction was rejected by the players.

Table 1 summarizes the experimental results for the objects submitted to the game for classification. The minimum number of matches for the bottles was obtained after 96 s, which triggered the pick-up. After that, labeling was still monitored until the 10 min mark for evaluation purposes. In Figure 8B, the matching rate of this experiment is compared to the expected values predicted by the proposed model and the matching constant *c*_*m*_ estimated in section 5.1. The actual matches over time are slightly lower than the prediction, but still closely match the trend. Compared to Figure 5A, the average number of matches is considerably lower than the corresponding case with five attributes. This is due to the increased matching difficulty, as 4 out of 5 object types in the bottle sorting experiment belonged to the same supercategory. Overall, the experiments demonstrated the potential of crowdsourcing through gamification in autonomous robotic environments, refining decision-making by employing human reasoning in the loop.

**Table 1:** Summary of results for object identification.

**Description**	**Value**
Can confidence	50.0%
Big bottle confidence	97.1%
Small bottle confidence	82.7%
Total matches	87 matches
Average bottle confidence	90.6%
Bottle confidence interval	80.7% < *p* < 95.6%

## 6. Discussion

The proposed attribute matching framework showed positive results in the evaluations, which indicate significant performance improvements with increasing crowd density. Operating with a limited number of label requests, a small fraction of the daily active player base could push labeling delays from minutes to near real-time performance. In addition to the crowd density, the framework performance depends on a number of other factors, including the game interface configuration, the number of submitted labels, the size of the attribute group, and the matching difficulty. These were captured through the proposed matching rate estimation model, which followed the actual matching activity in the live robot applications with a slight error. The discrepancy is likely a result of the chosen difficulty estimation method, which is based on the similarity of objects present in the game. This approach can not fully capture the matching difficulty, as it does not consider the object shape, background contrast, or image quality. The matching difficulty estimation could potentially be improved by incorporating a no-reference image quality assessment module, such as BRISQUE (Mittal et al., 2012).

An advantage of the proposed system is its flexibility, as it can be adapted and extended for any type of object attribute that a human can identify visually. Human perception is for instance unparalleled in the estimation of object affordances or “action possibilities” (Gibson, 1979; Montesano et al., 2008; Sun et al., 2010). Affordances play a major role in manipulation planning as they determine the appropriate grasp types for particular objects, which can be very beneficial for frameworks relying on vision (Zeng et al., 2018; Ficuciello et al., 2019). Visual estimation of object characteristics other than the class was presented through the exoskeleton glove experiments, where the chosen object attribute was its stiffness. Those experiments have also demonstrated the limits of visual attribute estimation in the case of cheese, where a consensus on its stiffness could not be reached.

An inherent limitation of the matching system is that every attribute requires a number of entries in the initial seed database. Since players can only match new images with ground truth examples, a label request can only be assigned an attribute that is already represented in the database. A solution for this would be to grant administrative rights to verified clients, allowing them to create and manage their own seed databases and attribute groups to fit their needs. Adding a new attribute to the seed database would, in this case, require only a small amount of annotating effort on the client side.

Another issue exposed in the experiments is the effect of attribute group sizes on labeling speed and accuracy. With increasing numbers of attribute classes and no prior predictions on label requests, the number of game instances where unknown tiles can be paired with the correct ground truth instances decreases. In many such cases, unknown tiles are mixed with tiles that are unrelated to them in terms of attributes, confusing the players and increasing the likelihood of incorrect matches. The objectively incorrect matches with unlabeled tiles get accepted by the game since they can not be verified, which reinforces the player to match the particular tiles in a wrong manner in the future. This can be effectively addressed by including label hints or permissible attribute classes into the label request. With an initial guess, the server would be able to create game parameters where attributes of known tiles are more likely to match with the unlabeled tiles. In addition, this issue would also be mitigated by larger crowds of participating players.

## 7. Applications

The proposed framework can be employed in two main application categories: real time robotic perception enhancement and passive database generation. The practical applications presented in this paper were examples of the former, although the labeling performance was not exceptionally responsive due to the relatively small group of participating players. Depending on the crowd density and the number of submitted label requests, this delay can range from seconds to minutes, which may not be sufficient for certain real time applications. However, the methodology can be efficiently integrated into systems that are able to postpone interaction with unknown objects. For instance, the framework can be effectively applied with indoor service robots that operate in a bounded environment. In such applications, the robot can request an attribute estimate as soon as a new, unknown object is encountered, even though it may not need to interact with it at that time. This allows the robot's perception system to gradually adapt to a changing environment through periodic re-training on newly labeled objects. Another example is autonomous waste sorting, where unrecognized objects or materials can be put aside until the appropriate attribute estimates are received. While waiting on the crowd consensus, the system can still manipulate objects that are recognized with the dedicated vision system, as was demonstrated in the bottle picking experiments. The second group of applications concerns passive database generation, where a client submits several label requests with the goal of expanding their attribute database. In this context, the framework does not need to support the operation of a live robot system, allowing such applications to request estimates of higher confidence at the cost of longer labeling times. This approach can be employed to create labeled collections from raw images available in public databases or obtained through mobile robot exploration in unknown environments.

The framework and game parameters should be configured with respect to the requirements of the target application. Since the number of active label requests directly affects estimation delays, real-time robot systems should aim to submit fewer labels at a time, while offline systems can afford to submit larger quantities. If permissible, real-time systems can also be configured with lower confidence thresholds to boost the estimation speed. The number of attribute classes should be kept as low as possible with a recommended maximum of 10, as the experiments have shown a significant drop in estimation confidence above this limit. If shrinking the number of attribute classes is not an option, the attribute group can be split into several smaller clusters of similar classes, which are linked to a parent group of attribute categories. The images can thus be labeled in a hierarchical manner through groups of appropriate size, providing attribute estimates with higher confidence. The server is designed to grant as much flexibility to the robot applications as possible, and will not attempt to adjust the client configuration in case of poor performance. Instead, the client applications can monitor the framework performance over time and employ the proposed matching rate estimation model to adjust their parameters with respect to the desired confidence and response time.

## 8. Conclusion and Future Work

This work proposed a crowdsourced attribute matching framework that enhances robot perception by leveraging human intelligence in grasping and manipulation tasks. Decisions of the participating crowd are collected through an online tile-matching game that is designed to entertain and motivate the players. The framework can identify unknown object attributes by linking them to a collection of ground truth images that expands through crowd participation. The system was evaluated in terms of matching rate and attribute estimation accuracy, with respect to the number of attribute classes and unknown labels. A model for estimating the expected matching rate was also proposed and validated. The framework was successfully employed in two proof-of-concept robotic applications, serving both as a primary attribute classification module and as a supplementary prediction refinement tool. It was shown that the small crowd of players was able to efficiently classify attributes of novel objects encountered by the robot, based on a compact database of seed images.

To ensure stability of the proposed framework, its security and robustness will need to be further considered. A large scale evaluation over a longer period of time will also be necessary to accurately estimate the expected response times with respect to the number of active players and label requests. This will enable the implementation of a load-balancing system to limit the volume of accepted label requests and ensure appropriate response times. This information could also be used in game level planning, to find the optimum balance between player experience, game difficulty, and labeling quality. In this process, game design aspects and theories will be taken into consideration.

Beyond the simple case of discrete label assignment, approaches for applying this methodology to find solutions to continuous problems will be investigated. These may include control of complex end-effectors, determining appropriate force profiles for object manipulation, or reactive control in collaborative tasks. Such tasks will likely demand user interfaces with higher flexibility, such as virtual or augmented reality devices (VR/AR). Through VR/AR environments, the robot's surroundings can be captured, encoded, and reconstructed in an immersive manner, giving the player a richer experience with more environmental detail. To tackle complex continuous problems, such interfaces can be intuitively integrated with motion capture devices tracking the user head, body, and hand motion.

## Data Availability Statement

The raw data supporting the conclusions of this article will be made available by the authors, without undue reservation.

## Ethics Statement

The studies involving human participants were reviewed and approved by the University of Auckland Human Participants Ethics Committee (UAHPEC) with the reference number #019043. The patients/participants provided their written informed consent to participate in this study.

## Author Contributions

GG contributed on the idea of the framework, conceived the structure for the final framework, and he developed the game, the framework, and the ROS package of the robotic platform. LG developed the vision based object identification code and conducted the robotics experiments. ML contributed on the idea and structure of the framework and supervised GG and LG in the implementation of the different components. The manuscript and videos were prepared by the authors collectively. All authors contributed to the article and approved the submitted version.

## Conflict of Interest

The authors declare that the research was conducted in the absence of any commercial or financial relationships that could be construed as a potential conflict of interest.
